# β-Hydroxy-β-methylbutyrate (HMβ) supplementation stimulates skeletal muscle hypertrophy in rats via the mTOR pathway

**DOI:** 10.1186/1743-7075-8-11

**Published:** 2011-02-23

**Authors:** Gustavo D Pimentel, José C Rosa, Fábio S Lira, Nelo E Zanchi, Eduardo R Ropelle, Lila M Oyama, Cláudia M Oller do Nascimento, Marco Túlio de Mello, Sergio Tufik, Ronaldo VT Santos

**Affiliations:** 1 Department of Physiology of Nutrition, Federal University of São Paulo (UNIFESP), São Paulo, SP - Brazil; 2 Department of Internal Medicine, FCM, State University of Campinas (UNICAMP), Campinas, SP - Brazil; 3 Department of Cell Biology and Development, Molecular Cell Biology Study Group - Institute o Biomedical Sciences I, University of São Paulo (USP), São Paulo, SP-Brazil; 4 Faculty of Applied Sciences, State University of Campinas (UNICAMP), Limeira, SP - Brazil; 5 Psychobiology & Biosciences, Federal University of São Paulo (UNIFESP), São Paulo, SP - Brazil; 6 Department of Bioscience, Federal University of São Paulo (UNIFESP), Baixada Santista Campus, São Paulo, SP - Brazil

## Abstract

β-Hydroxy-β-methylbutyrate (HMβ) supplementation is used to treat cancer, sepsis and exercise-induced muscle damage. However, its effects on animal and human health and the consequences of this treatment in other tissues (e.g., fat and liver) have not been examined. The purpose of this study was to evaluate the effects of HMβ supplementation on skeletal muscle hypertrophy and the expression of proteins involved in insulin signalling. Rats were treated with HMβ (320 mg/kg body weight) or saline for one month. The skeletal muscle hypertrophy and insulin signalling were evaluated by western blotting, and hormonal concentrations were evaluated using ELISAs. HMβ supplementation induced muscle hypertrophy in the extensor digitorum longus (EDL) and soleus muscles and increased serum insulin levels, the expression of the mammalian target of rapamycin (mTOR) and phosphorylation of p70S6K in the EDL muscle. Expression of the insulin receptor was increased only in liver. Thus, our results suggest that HMβ supplementation can be used to increase muscle mass without adverse health effects.

## Introduction

The amino acid leucine has been shown to stimulate skeletal muscle protein synthesis and attenuate muscle proteolysis. Some of these benefits have been attributed to the conversion of leucine to its metabolite β-hydroxy-β-methylbutyrate (HMβ) [1-4], which has been shown to be anti-catabolic and effective at attenuating muscle atrophy during exercise stress [2,5,6] and in models of cancer [4,7], congestive heart failure, sepsis, and HIV [8,9]. According to a review published by Nelo et al. [10], the dose of HMβ supplementation used in the majority of the previous studies to achieve these effects was 3 g/day of HMβ; use of this dose is based on evidence that it produces better results than 1.5 g/day.

HMβ is produced by the transamination of α-ketoisocaproate (KIC), which is metabolised to isovaleryl-CoA by the enzyme KIC dioxygenase. The cytosolic dioxygenase enzyme differs from the mitochondrial KIC dehydrogenase enzyme in several ways. KIC dioxygenase produces free HMβ in the cytosol, whereas the dehydrogenase enzyme produces the CoA derivative of isovaleric acid in the mitochondria [11,12]. Quantitatively, it is estimated that approximately 5% of all L-leucine oxidised in the human body is converted to HMβ. Therefore, a subject weighing 70 kg produces approximately 0.2 - 0.4 g HMβ/day, depending on the content of leucine in the diet. Because leucine, like all essential amino acids, is not synthesised in the human body, HMβ is produced from dietary protein [13].

The results of trials evaluating the effect of HMβ supplementation on reversing cachexia associated with rheumatoid arthritis [14], HIV [8], and muscle damage [15,16] are performed merely with biochemical markers. Additionally, most of the available studies concerning the effects of HMβ are related to effects on skeletal muscle, with an absence of relevant information concerning consequences in other peripheral tissues (e.g., adipose tissue and liver).

Recently, Holecek et al. [17] demonstrated increases in protein synthesis in the liver upon HMβ supplementation. However, the authors did not describe the mechanisms involved in this increase. Moreover, descriptions of the effects of HMβ alone (i.e., in normal situations without exercise or excess catabolism) are scarce.

Taking this into consideration, we aimed to examine the effect of HMβ supplementation on skeletal muscle hypertrophy in healthy and sedentary rats. The expression of the mammalian target of rapamycin (mTOR) and other proteins involved in insulin signalling were investigated to better understand HMβ-stimulated skeletal muscle hypertrophy.

## Methods

### Animals

The Experimental Research Committee of the Federal University of São Paulo approved all procedures for the care of the animals used in this study. A total of 14 male Wistar rats ranging in age from 8-9 wks and weighing between 200 and 250 g were used. They were housed four per cage, with a chow diet (NUVILAB) and water *ad libitum*, in an animal room with a 12 h light-dark cycle at 22 ± 1°C and 60 ± 5% humidity. The experiments were carried out after an acclimation period of one week.

### HMβ supplementation

Supplementation was carried out by intragastric administration (gavage) of 320 mg/kg body weight of HMβ (Dymatize Enterprises Inc, Dallas, TX, USA) diluted in 1.0 ml of water. HMβ was given daily at the same time (during the light period) for one month (at 3-4 months of age). This dose of supplementation was previously described by our group [4]. The control group was not submitted to the supplementation protocol and was treated with water by gavage.

### Blood glucose and lipidic profile measurements

Approximately 15-18 hours after HMβ oral gavage and after a 12 hour fast, the animals were euthanised by decapitation, blood was collected, and serum samples were collected after allowing the blood to clot on ice. Serum was stored frozen at -80°C for analysis. Labtest^® ^kits were used to assess fasting blood glucose, total cholesterol, high-density lipoprotein (HDL-c) and triacylglycerol (TG) levels. The samples were analysed using an enzymatic method. LDL-c and VLDL-c were calculated according to the Friedwald equation ((LDL-c = total cholesterol-(HDL-c)-(TG/5) and (VLDL = TG/5)) [18], and LDL-c subclass, which is a good predictive factor for oxidised-LDL-c, was calculated using the equation LDL-c = TG/HDL-c [19].

### Serum hormone levels

Serum fasting insulin, testosterone and corticosterone levels were quantified using enzyme-linked immunosorbent assay (ELISA). The insulin ELISA kit was obtained from Millipore Corp. (Bedford, MA, USA), and the testosterone and corticosterone kits were from Assay Designs, Inc. (Ann Arbor, MI, USA).

### Hepatic content of total lipids

The liver lipids were extracted according to the method of Folch [20].

### Protein analysis by western blotting

After euthanasia, the extensor digitorum longus muscle (EDL), retroperitoneal adipose tissue (RPAT), and liver tissues were rapidly removed and homogenised in 1.5 ml extraction buffer (100 mM Trizma, 1% SDS, 100 mM sodium pyrophosphate, 100 mM sodium fluoride, 10 mM EDTA and 10 mM sodium vanadate) and boiled for 10 min. The extracts were then centrifuged at 12,000 rpm at 4°C for 40 min to remove insoluble material. Supernatant protein concentration was determined using the Bradford dye method with a Bio-Rad reagent (Bio-Rad Laboratories, Hercules, CA, USA). The proteins were added to Laemmli sample buffer containing dithiothreitol and boiled for 5 min before loading onto 10% SDS-PAGE gels in a Bio-Rad miniature slab gel apparatus. Electrotransfer of proteins from the gel to nitrocellulose was performed for ~1 h at 15 V (constant) in a Bio-Rad semi-dry transfer apparatus. Nonspecific protein binding to the nitrocellulose was reduced by preincubation for 2 h at 22°C in blocking buffer (5% nonfat dry milk, 10 mM Tris, 150 mM NaCl and 0.02% Tween 20). The nitrocellulose membranes were separately incubated overnight at 4°C with antibodies against phospho-p70S6K, phospho-Akt, mTOR, GLUT-4, and Akt/PKB (Cell Signaling Technology^® ^(Danvers, MA, USA)) or AMPK, p70S6K, IR (insulin receptor) and alpha-tubulin antibodies, which were obtained from Santa Cruz Biotechnology (Santa Cruz, CA, USA), diluted in blocking buffer added with 1% bovine serum album (BSA) and then washed for 30 min in blocking buffer without BSA. The blots were subsequently incubated with peroxidase-conjugated secondary antibody for 1 h.

For evaluation of protein loading, membranes were stripped and reblotted with an anti-alpha-tubulin antibody. Specific bands were detected by chemiluminescence, and visualisation/capture was performed by exposure of the membranes to RX films. Band intensities were quantified by optical densitometry of developed autoradiographs (Scion Image software-Scion Corporation, Frederick, Md., USA).

### Statistical analysis

The statistical analysis was performed using the GraphPad Prism statistics software package version 5.0 for Windows (GraphPad Software, San Diego, CA, USA). The data are expressed as the means ± SEM. Implementation of the Kolmogorov-Smirnov test revealed that the results of experiments were distributed normally. The data were analysed using Student's t-test for comparison between two groups. A value of P < 0.05 was considered statistically significant.

## Results

### Body mass

HMβ treatment induced a significant increase in weight of the EDL and soleus muscles but did not change the total body weight, food intake, or fat and liver weight (Table 1).

**Table 1:** Body mass, food intake, and tissues weight of studied groups.

Variables	Control (n = 7) ME ± SEM	HMβ (n = 7) ME ± SEM	P Value
Weight (g)			-
Initial	306.10 ± 6.24	307.40 ± 3.65	-
Final	393.80 ± 11.98	409.60 ± 9.81	-
Variation (g)	87.70	102.20	0.163
Food intake (g)	22.01 ± 2.31	22.89 ± 2.43	0.402
EDL (g)	0.156 ± 0.002	0.162 ± 0.001	0.022*
Soleus (g)	0.177 ± 0.006	0.205 ± 0.011	0.024*
RPAT (g)	5.744 ± 0.870	4.624 ± 0.438	0.136
MEAT (g)	2.474 ± 0.225	2.207 ± 0.104	0.151
Liver (g)	11.56 ± 0.13	12.25 ± 0.41	0.110

EDL: extensor digitorum longus muscle, RPAT: retroperitoneal adipose tissue, MEAT: mesenteric adipose tissue.

*p < 0.05 vs. Control group.

### Glucose levels, lipidic profile, serum hormone levels and hepatic total lipid content

HMβ supplementation induced a significant increase in fasting insulin (+245%) and decreases in fasting glucose (-6%) and corticosterone (-48.7%). There were no differences in testosterone, lipidic profile, or hepatic total lipid content. Furthermore, an elevated (+65%) testosterone/corticosterone ratio was found in the HMβ group (Table 2).

**Table 2:** Hormones, serum, and hepatic content of total lipids parameters of control and HMβ groups.

Variables	Control (n = 7) ME ± SEM	HMβ (n = 7) ME ± SEM	P Value
Glucose (mg/dL)	148.9 ± 4.15	139.8 ± 2.36	0.047*
Insulin (ng/mL)	1.94 ± 0.85	4.75 ± 0.27	0.004*
Testosterone (ng/mL)	6.81 ± 0.38	7.14 ± 0.99	0.380
Corticosterone (ng/mL)	72.75 ± 5.15	37.32 ± 13.00	0.044*
Testosterone/corticosterone ratio	0.093 ± 0.014	0.154 ± 0.065	0.045*
Cholesterol (mg/dL)	177.3 ± 27.37	142.0 ± 13.69	0.166
Triacylglycerols (mg/dL)	169.0 ± 17.36	158.9 ± 8.24	0.327
HDL-c (mg/dL)	35.15 ± 6.18	45.22 ± 5.53	0.253
LDL-c (mg/dL)	152.00 ± 18.30	136.8 ± 12.48	0.265
VLDL (mg/dL)	33.80 ± 3.47	31.79 ± 1.64	0.327
Liver lipid (mg/dL 100 mg tissue)	72.56 ± 2.58	77.90 ± 3.80	0.578
TG/HDL-c ratio	5.64 ± 1.28	4.43 ± 0.12	0.209

HDL-c: high-density lipoprotein, LDL-c: low-density lipoprotein, VLDL-c: very-low-density lipoprotein, TG/HDL ratio: triacylglycerols/high-density lipoprotein ratio.

*p < 0.05 vs. Control group.

### Muscle protein synthesis (EDL)

**Figure 1:**
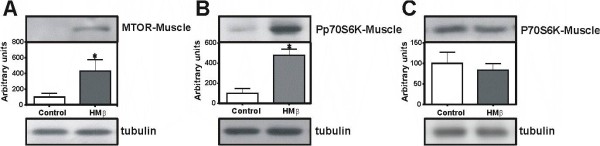
**The expression of mTOR (289 kDa) (A), phosphorylation of p70S6K (70 kDa) (B) and p70S6K (70 kDa) (C) proteins in the extensor digitorum longus (EDL) muscle after HMβ supplementation and control group by Western blotting**. N = 5 for all groups. Values are Mean ± SEM. * p < 0.05 vs. control.

HMβ supplementation induced a significant increase in mTOR expression (429.2%) (Figure 1A) and in phosphorylation of p70S6K (470%) (Figure 1B). However, p70S6K expression was not altered (Figure 1C).

### Protein expression involved in glucose uptake and insulin signalling

**Figure 2:**
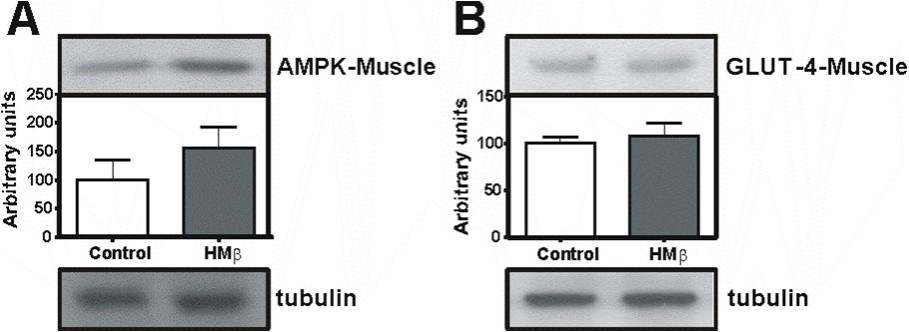
**The expression of AMPK (63 kDa) (A) and GLUT-4 (45 kDa) (B) proteins in the extensor digitorum longus (EDL) muscle after HMβ supplementation and control group by Western blotting**. N = 5 for all groups. Values are Mean ± SEM.

**Figure 3:**
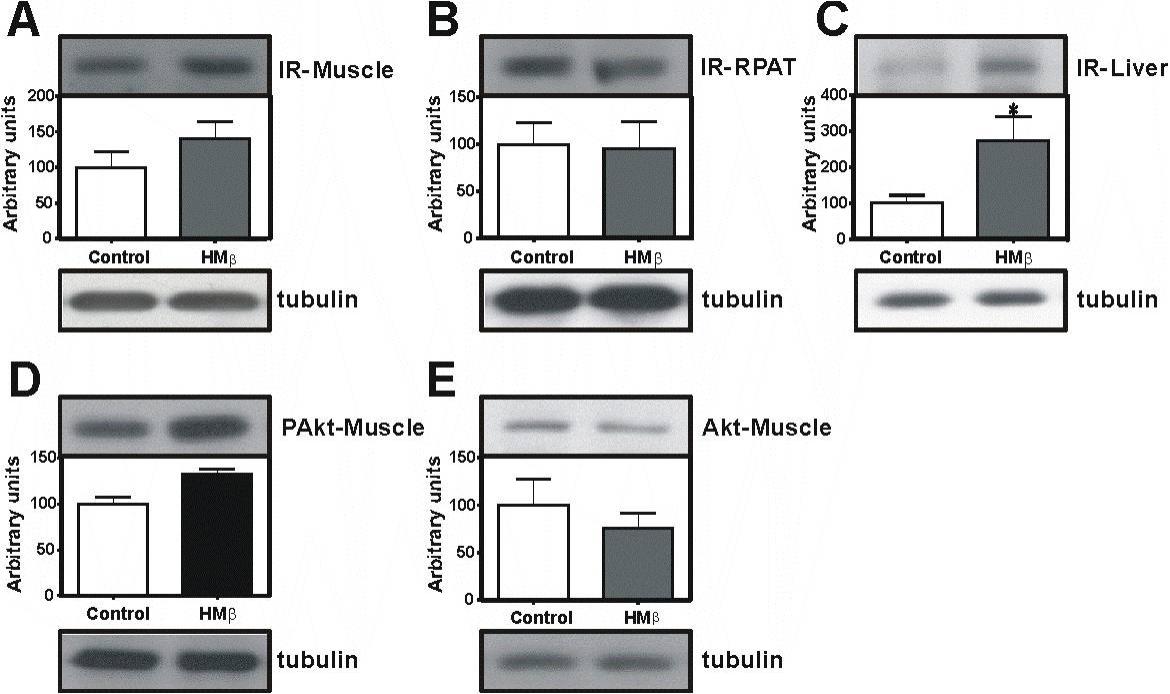
**The expression of IR (95 kDa) protein in the extensor digitorum longus (EDL) muscle (A), retroperitoneal adipose tissue (RPAT) (B) and liver (C), phosphorylation of Akt (60 kDa) (D) and Akt (E) after HMβ supplementation and control group by Western blotting**. N = 6 for all groups. Values are Mean ± SEM. * p < 0.05 vs. control.

AMPK and GLUT-4 levels were not altered with HMβ supplementation (Figure 2A and 2B, respectively). The following indicators of insulin signalling were evaluated: IR expression in the EDL muscle, RPAT and liver; as well as phosphorylation and expression of Akt/PKB in the EDL muscle. We observed that HMβ supplementation induced an increase in expression of IR only in the liver (272%) (Figure 3C). The relative expression levels of IR and Akt/PKB in the EDL muscle and in the RPAT were not altered (Figure 3A, B and 3E); the phosphorylation of Akt/PKB in the EDL muscle was also unchanged (Figure 3D).

## Discussion

The present study sought to evaluate the impact of HMβ supplementation on the expression of proteins involved in skeletal muscle hypertrophy in healthy and sedentary rats and proteins involved in insulin signalling. Our results show that HMβ supplementation increased mTOR expression and phosphorylation of p70S6K in the EDL muscle while increasing fasting insulin levels and testosterone/corticosterone ratios and decreasing fasting glucose and corticosterone levels in the serum.

As previously described, HMβ is a widely studied metabolite of leucine. Several reports have shown that branched-chain amino acids (BCAA), isolated leucine and HMβ can stimulate skeletal muscle protein synthesis and activate the mTOR pathway in skeletal muscle [9,21] as well as in primary hepatocytes [22].

In the present study, we observed that, relative to the control group, the supplemented group demonstrated an increase in mTOR protein levels and activation of p70S6K, which are linked to increased skeletal muscle mass in the EDL muscle. Our findings are supported by a recent study by Lang's group [23] showing that gastrocnemius mass and protein synthesis were robustly decreased in mTOR heterozygous mice compared to wild type mice. Based on this information, we conclude that not only the activity but also the level of mTOR is an important regulator of skeletal muscle mass.

Contrary to what was observed in the present study, Ostaszewski et al. [24] and Holecek et al. [17] did not observe increased protein synthesis in the EDL and soleus muscles after HMβ supplementation but measured consistently decreased protein degradation, as estimated by the net release of tyrosine from incubated muscles.

In the present study, we analysed the expression of AMPK, which is known to be an important regulator of muscle protein synthesis [25,26], but we found no differences between the groups.

In the present study, we found no alterations in the Akt/PKB pathway in the EDL muscle. Thus, we suggest that increased skeletal muscle protein mass by HMβ supplementation was induced directly via increased mTOR expression and activation of p70S6K and not via phosphorylation of Akt/PKB. However, as previously shown in several studies, constitutive activation of Akt/PKB is capable of inducing skeletal muscle hypertrophy [27-29], although we did not observe this effect in our study. Moreover, as the molecular analyses were performed 15-18 hours after HMβ oral gavage, we suggest that the activation of the mTOR/p70S6K pathway persists for many hours after supplementation. However, Laymans group has shown that peak mTOR and insulin signaling responses occur shortly after consumption of a meal (i.e. 1-3 hrs) [30]; considering that our measures were taken 15-18 hrs after the HMβ gavage, and after an overnight fast, it is possible that we missed certain signals. Therefore, the acute (1-3 hrs post gavage) effects of HMB on translation initiation and elongation factors, as well as insulin signaling warrant further investigation.

In the current study, we investigated the intracellular signalling pathways involved in increased protein synthesis induced by HMB; however, it is important to remember that potent hormones are secreted in response to nutrients and might exert robust increases in protein synthesis and metabolism in several tissues [31-33]. In the present study, we observed that increased insulin levels may have resulted in increased mTOR levels and phosphorylation of p70S6K; however, we have no data that directly address this idea, and previous studies [30,34] do not support the idea that typical fasting insulin concentrations (above basal levels) can stimulate the mTOR/p70S6K pathway.

As discussed above, several studies [16,22,26] have been performed to determine the main mechanism of action of HMβ. Based on the results of this study, we have outlined the main mechanisms of HMβ action as being related to increases in mTOR/p70S6K pathway signalling and most likely leading to improved protein synthesis and muscle hypertrophy. However, it is important to note that protein synthesis pathways are extremely redundant, and other important proteins not evaluated in our study, such as the eukaryotic translation initiation factor 4E (eIF4E/2B), might play a role in the observed final response (i.e., muscle hypertrophy).

In accordance with this idea, hormones control anabolic/catabolic pathways that can favour (insulin, testosterone) or antagonise (glucocorticoid hormones) [35,36] anabolism in skeletal muscle. In fact, we observed low serum corticosterone levels and high testosterone/corticosterone ratios after HMβ supplementation, which could contribute to skeletal muscle hypertrophy. Likewise, Olza et al. [37] observed that in elderly patients, a protein-enriched diet was able to increase protein synthesis and reduce protein degradation. Moreover, the reduction in corticosterone and increase in insulin levels may have favoured the reduction in fasting blood glucose after HMβ supplementation. Recently, Guo et al. [38] also found improvements in glucose homeostasis after leucine supplementation.

Hepatic lipid levels were not altered, as shown in Table 2. These results are consistent with the lack of change in serum lipid levels. Several studies [3,17] have shown that HMβ is metabolised to HMG-CoA and used for *de novo *synthesis of cholesterol in certain tissues, including the liver. However, this conclusion is debatable; Holecek et al. [17] showed increases in serum cholesterol levels after HMβ administration, while Nissen and Abumrad [3] showed decreases in LDL-c.

In the present study, we also demonstrated an increase in skeletal muscle weight (EDL and soleus) in the absence of changes in total body mass, fat mass or liver weight. However, is suggesting that other body measures of body fat, such as the size of different fat deposits, e.g., epididymal fat, may have changed.

As stated previously, the effects of HMβ supplementation in peripheral tissues have not been intensely investigated. In accordance with this, Pedrosa et al. [39] showed that leucine supplementation increased liver protein content. However, in this study, we thoroughly investigated HMβ's effects on proteins involved in insulin signalling, starting with the insulin receptor (IR), and showed that HMβ supplementation stimulated IR expression in the liver, likely due to mTOR activation. However, the effects of long-term HMβ supplementation on this outcome are as yet unknown.

In summary, HMβ treatment leads to skeletal muscle hypertrophy via increases in mTOR expression and decreases in serum corticosterone concentrations. In addition, HMβ supplementation increases IR expression in the liver compared to control group. Thus, our results suggest that HMβ supplementation can be used to increase muscle mass without adverse health effects.

## Conflicts of interests

The authors declare that they have no competing interests.

## Authors' contributions

GDP, JCR and FSL participated of sample collected, assess samples, design of the study and performed the statistical analysis, and writing of paper, NEZ and ERR helped carry out design of the study, LMO, CMON, MTM, ST and RVTS helped in design of the study and discussion of paper. All authors read and approved the final manuscript.
